# The comparison of biodistribution of glutathione PEGylated nanoliposomal doxorubicin formulations prepared by pre‐insertion and post‐insertion methods for brain delivery in normal mice

**DOI:** 10.1049/nbt2.12111

**Published:** 2023-01-03

**Authors:** Amin Mehrabian, Saba Dadpour, Mohammad Mashreghi, Javad Zarqi, Anis Askarizadeh, Ali Badiee, Leila Arabi, Seyedeh Alia Moosavian, Mahmoud Reza Jaafari

**Affiliations:** ^1^ Department of Pharmaceutical Nanotechnology School of Pharmacy Mashhad University of Medical Sciences Mashhad Iran; ^2^ Nanotechnology Research Center Pharmaceutical Technology Institute Mashhad University of Medical Sciences Mashhad Iran; ^3^ Nanotechnology Research Center Student Research Committee Faculty of Pharmacy Mashhad University of Medical Sciences Mashhad Iran

**Keywords:** brain, drug delivery systems, nanomedicine, nanoparticles

## Abstract

Several obstacles limit the efficacy of brain tumour treatment, most notably the blood‐brain barrier (BBB), which prevents the brain uptake of the majority of accessible medicines due to tight junctions. The presence of glutathione (GSH) receptors on the BBB surface has been demonstrated in numerous papers; consequently, products containing glutathione as a targeting ligand coupled with nanoliposomes are used to enhance drug delivery across the BBB. Here, the 5% pre‐inserted PEG2000‐GSH PEGylated liposomal doxorubicin was conducted according to 2B3‐101 being tested in clinical trials. In addition, PEGylated nanoliposomal doxorubicin connected to the spacer‐GSH targeting ligand (GSGGCE) and the PEG3400 was conducted using post‐insertion method. Next, in vivo biodistribution of the produced formulations was tested on healthy mice to see if GSGGCE, as the targeted ligand, could cross the BBB compared to 5% pre‐inserted PEG2000‐GSH and Caelyx^®^. Compared to the pre‐inserted formulation and Caelyx^®^, the post‐inserted formulations' concentration was lower in the heart and higher in brain tissues, resulting in boosting the brain concentration of accumulated doxorubicin with fewer possible side effects, including cardiotoxicity. In comparison to the pre‐insertion procedure, the post‐insertion method is easier, faster, and more cost‐effective. Moreover, employing PEG3400 and the post‐insertion approach in the PEG3400‐GSGGCE liposomal formulations was found to be effective in crossing the BBB.

## Graphical abstract

The 5% pre‐inserted PEG2000‐GSH PEGylated liposomal doxorubicin and PEGylated nanoliposomal doxorubicin connected to the spacer‐GSH targeting ligand (GSGGCE) and the PEG3400 was conducted using post‐insertion method. Next, biodistribution of the produced formulations was tested on healthy mice. Compared to the pre‐inserted formulation and Caelyx^®^, the post‐inserted formulations' concentration was lower in the heart and higher in brain tissues, resulting in boosting the brain concentration of accumulated doxorubicin with fewer possible side effects.

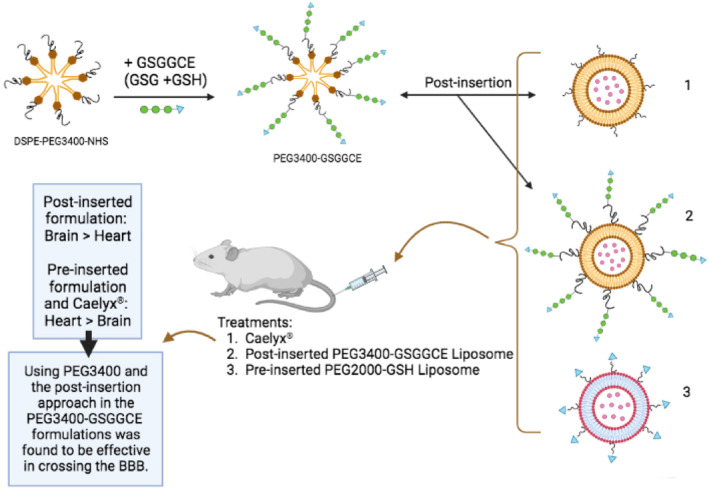

## 1 INTRODUCTION

Brain cancer is considered one of the most aggressive types of tumours and one of the most difficult‐to‐treat malignancies due to the presence of the physical and metabolic barrier known as the blood‐brain barrier (BBB) [1]. According to the American Cancer Society, malignant brain tumours were identified in 24,530 persons in the United States in 2021. Furthermore, through the end of 2022, it is anticipated that 18,280 adults will die from primary malignant brain and central nervous system (CNS) tumours [2]. Surgery, radiation, and chemotherapy, either alone or in combination, have been the gold standard in the treatment of brain cancer for the past decade [3]. Surgical resection is the most common treatment for brain tumours, which can help to improve survival rates and quality of life. However, due to tumour heterogeneity, complexity, and severity, it is not always a practical approach [4]. Radiation therapy is also commonly used after surgery to eliminate remaining tumour cells and alleviate illness symptoms. However, the precision of this approach is limited since it may cause radiation tissue damage in healthy brain tissues surrounding the tumour mass [5]. Furthermore, due to the presence of the BBB, chemotherapy is challenging and has some limitations. As a result, techniques to increase anti‐cancer therapy localised to the tumour site are required [6]. Nanomedicine has lately been regarded as a promising field for overcoming the limitations and flaws of existing drug delivery technologies [7]. Caelyx^®^, also known as PEGylated liposomal doxorubicin (PLD), is a nano carrier‐based medication used to treat a range of malignancies. When compared to the old form of doxorubicin (Dox), Caelyx^®^nanoliposomal structures have a better toxicity profile and efficacy profile [8, 9]. Additionally, nanoliposomal PEGylation enhances tumour accumulation by increasing the stability and blood circulation period [10].

As previously stated, despite advances in chemotherapeutics, overcoming BBB is still a challenge in brain tumour chemotherapy, which impedes the entrance of the therapeutic level of drugs into tumour tissues [11, 12]. Most macromolecules can cross the BBB through the receptors found on the BBB outer layer by receptor‐mediated transcytosis [13]. Nanocarriers coated with ligands have been proven in previous investigations to increase receptor‐mediated uptake and, as a result, drug transport into the brain [14, 15, 16].

Glutathione (GSH), an antioxidant tripeptide made up of three amino acids (cysteine, glutamic acid, and glycine), has been shown to protect the CNS from damage [17, 18]. Given the presence of glutathione receptors on the BBB, it stands to reason to use glutathione as a targeting ligand coupled with nanoliposomes [19, 20]. GSH‐functionalised PEGylated liposomal DOX (2B3‐101) is a promising GSH‐targeted liposome which has finished the phase I/IIA clinical trial for the treatment of brain cancer, demonstrating that DOX can be administered more efficiently to brain tumours [21, 22]. 2B3‐101 was prepared by incorporation of DSPE‐PEG2000‐Maleimide‐GSH into the existing product, Caelyx^®^. In this case, a 5% pre‐inserted PEG2000‐GSH PEGylated liposomal DOX with the same properties as 2B3‐101 was created. The pre‐insertion method used to generate the aforementioned formulations is not cost‐effective or technically feasible for ligand targeting [23]. In contrast, the post‐insertion procedure used in this study is thought to be a simpler, faster, and more cost‐effective strategy [24]. Furthermore, the post‐insertion method for preparing ligand‐targeted liposomes has been extensively studied by our team. The results showed that this method is simple, cost‐effective and reproducible when generating actively targeted liposomal DOX [25, 26, 27]. Similarly, in our previous work, using the post‐insertion method, we demonstrated that GSH‐targeted liposomes penetrated the BBB efficiently [28].

This method begins with the creation of lipid‐derivatised peptide micelles, which is then followed by the transfer of peptide‐coupled PEG‐lipid micelles to preformed liposomes, converting ordinary liposomes to targeted liposomes in a single step. This process successfully converts commercially available liposomal DOX (Caelyx^®^) to targeted sterically stabilised liposomal DOX. For future clinical development, the post‐insertion approach appears to be quite promising [29, 30, 31]. In the previous study conducted by our team, using 200 and 400 GSH ligands applied on the available Caelyx^®^ via the post‐insertion method could improve crossing through the BBB and brain biodistribution [28].

The purpose of this experiment was to achieve a high‐throughput method to address the challenges of targeted liposomes optimisation. Here, we tried to optimise the GSH‐targeted liposome platform and validate the effect of the functionalisation method on the biodistribution of liposomes in a preclinical approach. For this, we added the glycine‐serine‐glycine (GSG) sequence as a spacer and PEG3400 for incorporation of GSH on the surface of liposomes. Drawing on our previous findings as well as those of others, we hypothesised that the addition of longer spacers provides more recognition by the receptors. The ligand peptide was chosen because glutathione binds to its receptors expressed on the BBB. We also chose PEG3400 as the targeting moiety's linker because it has a longer spacer that optimises targeting ligand exposure to the BBB's GSH receptors [32].

PEG3400‐GSGGCE targeted liposomes were prepared using the post‐insertion method, which is regarded to be a simpler, faster, and less expensive method of preparing targeted liposomes than pre‐insertion. The characteristics and biodistribution of PEG3400‐GSGGCE targeted liposomes were compared to those of 5% pre‐inserted PEG2000‐GSH and commercially available Caelyx^®^ in healthy Naval Medical Research Institute (NMRI) mice. The designed post‐inserted formulations were equivalent to the 2B3‐101, which Gaillard et al. evaluated and proved earlier, based on their accumulation in brain tissue and less distribution to the heart, thus enhanced effectiveness and reduced cardiotoxicity [23].

## 2 METHODS AND MATERIALS

### 2.1 Materials

Avanti Polar Lipids provided methoxy‐polyethylene glycol (MW 2000)–distearoylphosphatidylethanoloamine (mPEG 2000–DSPE) (Alabaster, AL). Maleimide PEG2000 di stearoyl phosphatidylethanolamine or DSPE‐PEG (2000) Maleimide was purchased from Avantipolar (Alabaster, AL). Furthermore, Nanocs Inc. provided 1,2‐Distearoyl‐sn‐glycero‐3‐phosphoethanolamine‐N‐ [succinimidyl (polyethylene glycol)] (DSPE–PEG3400 NHS) (USA). Merck supplied the ‐L‐Glutamyl‐L‐cysteinyl‐glycine (L‐Glutathione). Behestan Darou and Sigma‐Aldrich provided Caelyx^®^ and Doxorubicin HCl (DOX) respectively. Merck provided the isopropanol, which was acidified by adding 7.5 ml HCl 1M and 2.5 ml water to 90 ml isopropanol. The chemical grade was applied to all other reagents and solvents.

### 2.2 Pre‐inserted formulation preparation

#### 2.2.1 PEG2000‐GSH preparation

The GSH peptide was dissolved in dimethyl sulfoxide (DMSO) and added to the DSPE‐PEG (2000) Maleimide chloroform solution to covalently attach it to the DSPE‐PEG (2000) Maleimide. The peptide to maleimide molar ratio was 1.2:1, and the volumetric ratio of DMSO to chloroform was 1:1. For the reaction, they were constantly mixed at 37°C for 48 h. Finally, the solvents were removed and freeze‐dried using a rotary evaporator and a freeze dryer (Heidolph) (VD‐800F).

#### 2.2.2 PEG2000‐GSH evaluation

Thin‐layer chromatography was used to evaluate the freeze‐dried product after it was dissolved in ammonium sulphate (TLC). With a 45/9/1 chloroform/methanol/water mobile phase ratio and iodine vapour exposure, the peptide‐lipid conjugate was observed on a TLC plate.

Furthermore, reverse‐phase liquid chromatography was used to assess the peptide reaction with PEG (Shimadzu). The compound was tested using 0.001 phosphoric acids high‐performance liquid chromatography (HPLC) grade water as the mobile phase in an isocratic gradient. Free GSHs were added to the final product without any maleimide reactive groups for technique validation, and they were handled the same as the others.

#### 2.2.3 Pre‐insertion method

Stock lipid solutions of hydrogenated soy phosphatidylcholine and cholesterol, as well as the prepared PEG2000‐GSH, were mixed in a round bottom flask to make the 5% pre‐inserted PEG2000‐GSH PEGylated liposomal DOX. A rotary evaporator was used to extract the solvent under reduced pressure, resulting in a thin film on the flask wall. The solvent residues were removed using a freeze‐dryer connected to a vacuum pump. The lipid film was then hydrated in 250 mM ammonium sulphate buffer at 65°C) for 30 min, vortexed for 30 min, and then sonicated in a water bath (68°C). The milky white suspension was frozen and thawed three times before extrusion. A single freeze‐thaw cycle consisted of freezing at liquid nitrogen (−196°C) and thawing at 65°C in a water bath. The suspension was extruded 11 times through polycarbonate membranes using the LIPEX^TM^ extruder to make liposomes. These procedures were carried out at a temperature of 65°C. In order to exchange ammonium sulphate with histidine buffer, the produced liposomes were dialysed three times using a 12–14 KDa molecular weight cut‐off (MWCO) (pH 6.5). The phospholipid content of the liposomal formulation was measured in the last phase using the Bartlett phosphate test. The required DOX concentration was added to the liposome for remote loading of DOX, and they were co‐incubated at 65°C for 1 h with gentle shaking, then purified by dialysis (12–14 KDa MWCO) against dextrose‐histidine buffer.

### 2.3 Post‐inserted formulations preparation

#### 2.3.1 PEG3400‐GSGGCE preparation

The described liposomes were prepared using the post‐insertion method. In a nutshell, DSPE–PEG3400 NHS was mixed in a 4:1 M ratio with mPEG2000–DSPE and dried under N2 streams. mPEG2000–DSPE was required to avoid intermicellar crosslinking during coupling with the peptide and DSPE–PEG3400–NHS as a linker [33]. The dried lipid film containing DSPE–PEG3400–NHS and mPEG2000–DSPE was hydrated in sterile water at 47°C before being mixed with the peptide (GSGGCE) at a 10:1 M ratio in phsphate buffered saline (PBS) (pH 7.4) [34].

#### 2.3.2 PEG3400‐GSGGCE evaluation

The result of this reaction was determined using HPLC to validate the binding of the GSGGCE peptide to the DSPE‐PEG3400‐NHS phospholipid. In this regard, from a reverse‐phase column C18, with a diameter of 4.6 mm, a length of 250 mm was used. At 220 nm, chromatograms were collected. The mobile phase flow rate was 1 ml/min, and the column temperature was 30°C. The solvent system is comprised of two different polarity solvents: acetonitrile with 0.1% trifluoroacetic acid (solvent A) and deionised water with 0.1% trifluoroacetic acid (solvent B) (solvent B). These solvents and conditions were in accordance with the HPLC program of the peptide provider's instructions. The schedule for changing the proportion of solvents passing through the chromatogram column for GSGGCE peptide, according to the peptide manufacturer's protocol, is as follows: first within 20 min, solvent system A from 1% to 20%, and then within 20 min, solvent system B from 99% to 80%. Then, in less than 0.1 min, system solvent A surged from 20% to 100%, while system solvent B decreased from 80% to 0%. The program's overall cycle time was 20 min. Peptide‐lipid and free peptide chromatograms were taken from the column at different times that reached the detector under these settings.

#### 2.3.3 Post‐insertion method

The PEG3400‐GSGGCE micelles were incubated with commercially available Caelyx^®^ for 1 h at 60°C with gentle shaking. The dialysis technique was utilised to remove free peptides from liposomes before HPLC analysis using a 12‐KDa MWCO dialysis membrane. To develop the PEG3400‐GSGGCE liposomal formulations, 100, 200, and 400 ligands were placed on the liposomal surface. The total number of peptide molecules per liposome was determined using the following parameters: (a) Caelyx^®^ phospholipids concentration; (b) liposomal average size; (c) lipid molecules per liposome with the average size; (d) liposomal numbers per 1 ml; (e) total peptide content; (f) peptide molecules per 1 ml of peptide‐micelles; and (g) the number of peptides per liposome [24].

### 2.4 Characterisation of liposomes

A Dynamic Light Scattering (DLS) instrument was used to analyse parameters such as particle size, zeta‐potential, and polydispersity index (PDI) of the liposomal formulations produced (Nano‐ZS; Malvern). HPLC was also used to determine the phospholipid content of the targeting ligands. The encapsulation efficiency (EE%) of each formulation was assessed by measuring the DOX concentration with a spectrofluorometer (Shimadzu RF5000U (ex: 480 nm/em: 580 nm), before and after pre‐ and post‐insertion steps via dialysis, and compared with that of Caelyx^®^ [35]. Furthermore, evaluation of the produced liposomes' morphological characteristics was done using transmission electron microscopy (TEM). In order to prepare the samples for TEM, a drop of each diluted PLD was applied to a carbon‐coated copper grid, surplus samples were then removed, and a drop of uranyl acetate was applied to the grid for negative staining. Finally, using a LEO 912 TEM with an acceleration voltage of 80 kV, the samples could be viewed (Zeiss) [36].

### 2.5 Drug release study

Each formulation was placed in a dialysis bag (cut off 12–14 KDa), immersed in three separate media with pHs of 5.5 (succinate), 6.5 (histidine), and 7.4 (PBS), and incubated overnight at 37°C. Spectrofluorometer (ex: 490 nm/em: 585 nm) was used to determine the amount of released DOX collected from the release media at predetermined time points over 48 h [37].

### 2.6 Animal study

Female NMRI mice aged 8–10 weeks were purchased from the Pasteur Institute.

The animal studies followed Mashhad University of Medical Sciences' Institutional Ethical Committee and Research Advisory Committee norms. All of the microenvironments (cage, food, water, and cleaning), as well as the macro environments (temperature and light/dark cycle), were given in accordance with the standards.

### 2.7 Biodistribution study

The produced peptide‐PEG micelles were subsequently inserted on Caelyx^®^ nanoliposomes at 60°C for 1 h before injection. The animals were then given a single dosage of pre‐ and post‐inserted formulations via the tail vein. At 6‐, 24‐, and 48‐h following injection, blood samples were taken through retro‐orbital bleed.

The mice were euthanised 48 h after receiving the formulations, and blood samples were obtained via heart puncture. Organs such as the brain, spleen, lungs, kidneys, heart, and liver were separated, weighed, and placed in 2 ml polypropylene micro vials containing 1 ml acidified isopropanol and zirconia beads, before being homogenised with a Mini‐Beadbeater (Biospec). All blood and organ samples were kept at 4°C overnight before proceeding to the next procedure. They were then centrifuged for 10 min at 14,000 rpm, and the supernatants were evaluated using a spectrofluorometer (ex: 490 nm, em: 590 nm).

Serial dilutions were used to create the calibration curve [38].

### 2.8 Histological study

The mice were sacrificed 24 h after receiving each formulation (10 mg/kg) in order to conduct the histopathological study. Their brains were then extracted, embedded in paraffin, and stained with fluroushiled™ with DAPI (Sigma‐Aldrich). Finally, the DOX permeation into the brain tissues was assessed using fluorescence microscopy [39].

### 2.9 Statistical analysis

The statistical analysis was performed using GraphPad Prism version 6 (GraphPad Software). The significance of the differences between the groups was determined using a two‐way analysis of variance (ANOVA) with Tukey's post‐test. Statistical significance is defined as a *p*‐value of less than 0.05 (*p* < 0.05).

## 3 RESULTS

### 3.1 Characterisation of PEG2000‐GSH

TLC and HPLC were used to examine the final conjugation product to ensure that the linking reaction was completed successfully. TLC showed the unconjugated peptide and lipid plus the conjugation reaction product due to differing mobility on silica gel paper (Figure S1). PEG has moved further, as expected, whereas the PEG2000‐GSH combination and peptide have remained immobile. No spots related to PEG migration were discovered, implying a nearly perfect linking efficacy in the conjugation reaction.

Figure S2 shows the HPLC chromatography of the free peptide and the final product based on retention time to confirm the final product and the linking reaction. The free peptide was initially evaluated, and the graph's peak was created (peak A). Similarly, a final conjugation product was put into the HPLC column, and the complex graph was achieved 7 min after injection (peak B). Finally, a mixture of free peptide and complex was injected to confirm and identify the curves displayed (peak C).

### 3.2 Characterisation of PEG3400‐GSGGCE

In the case of GSGGCE peptide, a specific concentration of free peptide in an aqueous solution and PBS was first reacted with DSPE‐PEG3400‐NHS in the above proportion. Prior to injection of the reaction product, a free peptide peak was obtained with a retention time of 2.11 min (Figure S3A). Then, the reaction product was injected, and the corresponding peak was obtained (Figure S3C). By holding peaks A and C under which the peptide with a particular concentration before the reaction and the free peptide did not react in the final solution after the reaction (peak C), respectively, the concentration of free peptide in the final solution can be obtained.

theareaunderthepeakAareunderthepeakC=freepeptidebeforereaction/freepeptideafterthereaction

The exact number of micrograms per millilitre of the reactive peptide can be obtained by decreasing the concentration of free peptide in the final reaction solution from the initial amount before the beginning of the reaction. By holding the molecular weight of the peptide, which is 508.5 g per mole, it is possible to obtain the nanomole of the peptide, therefore, the product obtained in the produced microlitre. In fact, this conclusion is the result of the fact that for each peptide number, an amide bond is formed and a product number is present. Next, the number of target ligands for this study is 100, 200, and 400 ligands; by considering the number of moles of phosphate per mole of the product which is 1: 1 and multiplying by the Avogadro number, the required number of molecules for the mentioned formulas can be obtained.

### 3.3 Characterisation of pre‐ and post‐inserted formulations

**FIGURE 1:**
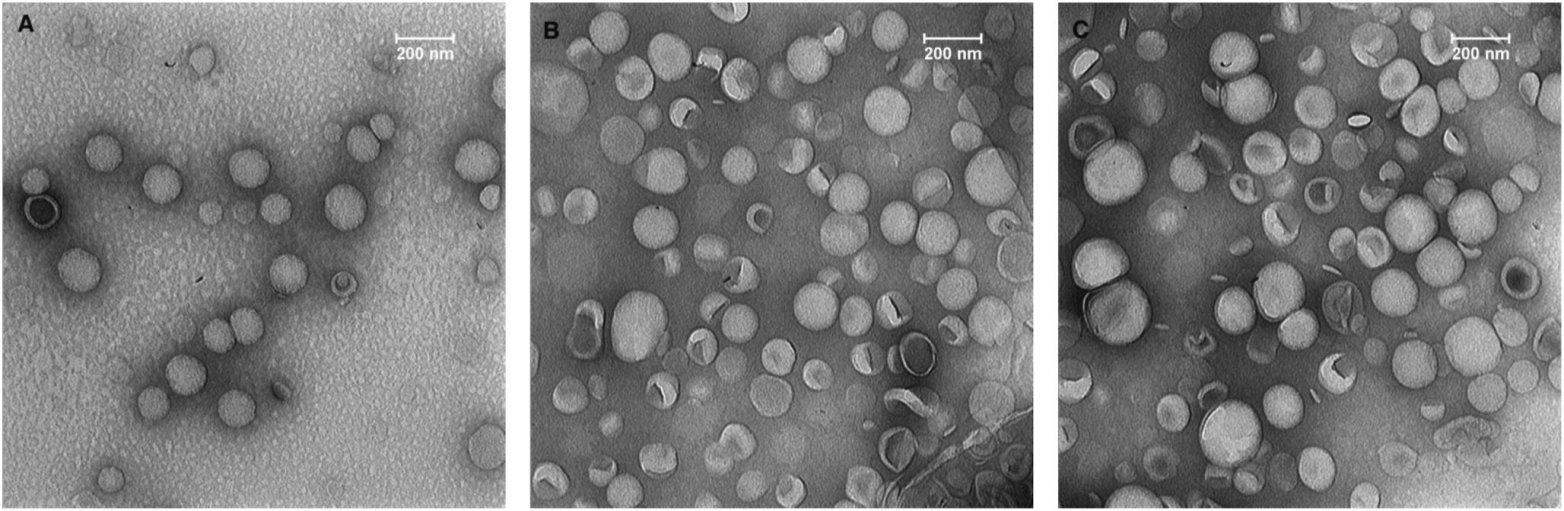
Transmission electron microscopy (TEM) images of GSH‐PEG liposomal formulations. (a) 400L post‐inserted formulation, (b) 5% pre‐inserted formulation, (c) Caelyx®.

Dynamic Light Scattering or DLS evaluated parameters such as particle size, PDI, and zeta potential in liposomal formulations. As demonstrated in Table 1, all of the post‐inserted formulations, as well as Caelyx^®^, were smaller than 100 nm in diameter, which is suitable for intravenous delivery. The particle size of the 5% pre‐inserted formulation, on the other hand, was greater than 100 nm. In all formulations, the PDI, which measures particle homogeneity, was less than 0.2 [40]. The zeta potential of the post‐inserted formulations was negative (∼ −15 mV), similar to Caelyx^®^. The zeta potential of the 5% pre‐inserted formulation was lower than the zeta potential of the other formulations (−20.0 ± 0.6 mV). The encapsulation efficacy was measured after pre‐ and post‐insertion, and it was greater than 95% for all of the formulations except the 5% pre‐inserted formulation, which was almost 90%. Moreover, the TEM images (Figure 1) confirmed that the generated PLD formulations were almost uniform and spherical in shape, with diameters of about 100 nm, which was consistent with the DLS data presented in Table 1.

**TABLE 1:** Physicochemical characteristics of the pre‐ and post‐inserted formulations and Caelyx®

Treatments	*Z*‐average size (nm)	PDI	Zeta potential (mV)	DOX remained in liposomes after pre‐ and post‐insertion (%)
100L	98.6 ± 0.2	0.1 ± 0.0	−15.2 ± 0.7	98.0 ± 1.2
200L	98.3 ± 1.2	0.1 ± 0.0	−15.5 ± 0.6	96.0 ± 1.8
400L	101.7 ± 0.7	0.1 ± 0.1	−15.3 ± 0.8	96.0 ± 2.1
5%	109.7 ± 0.3	0.2 ± 0.0	−20.0 ± 0.6	90.0 ± 5.3
Caelyx®	94.7 ± 1.7	0.2 ± 0.1	−15.1 ± 0.5	97.0 ± 1.1

*Note*: Data are reported as mean ± standard deviation of three independent preparations.

### 3.4 Drug release

**FIGURE 2:**
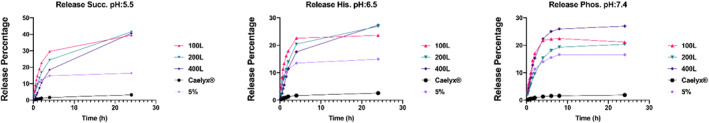
The release profile of the pre‐ and post‐inserted formulations and Caelyx® at different pHs of 5.5 (succinate buffer), 6.5 (histidine buffer), and 7.4 (phosphate buffer).

To mimic physiological, tumoral, and endosomal circumstances, the release profile as a measurement of stability, was evaluated at varied pHs (7.4, 6.5, and 5.5) [41]. Caelyx^®^ had a release of less than 2%, which was significantly lower than the other formulations at all pHs, as shown in Figure 2. Furthermore, at all pHs, there was no discernible variation in the release profiles of the post‐inserted formulations. In the first 10 h, they all trended higher, and their release profiles did not vary significantly during the next several hours. The release of the 5% pre‐inserted formulation, on the other hand, was much lower than that of the post‐inserted formulations, but significantly higher than that of Caelyx^®^, with a similar trend.

### 3.5 Biodistribution

**FIGURE 3:**
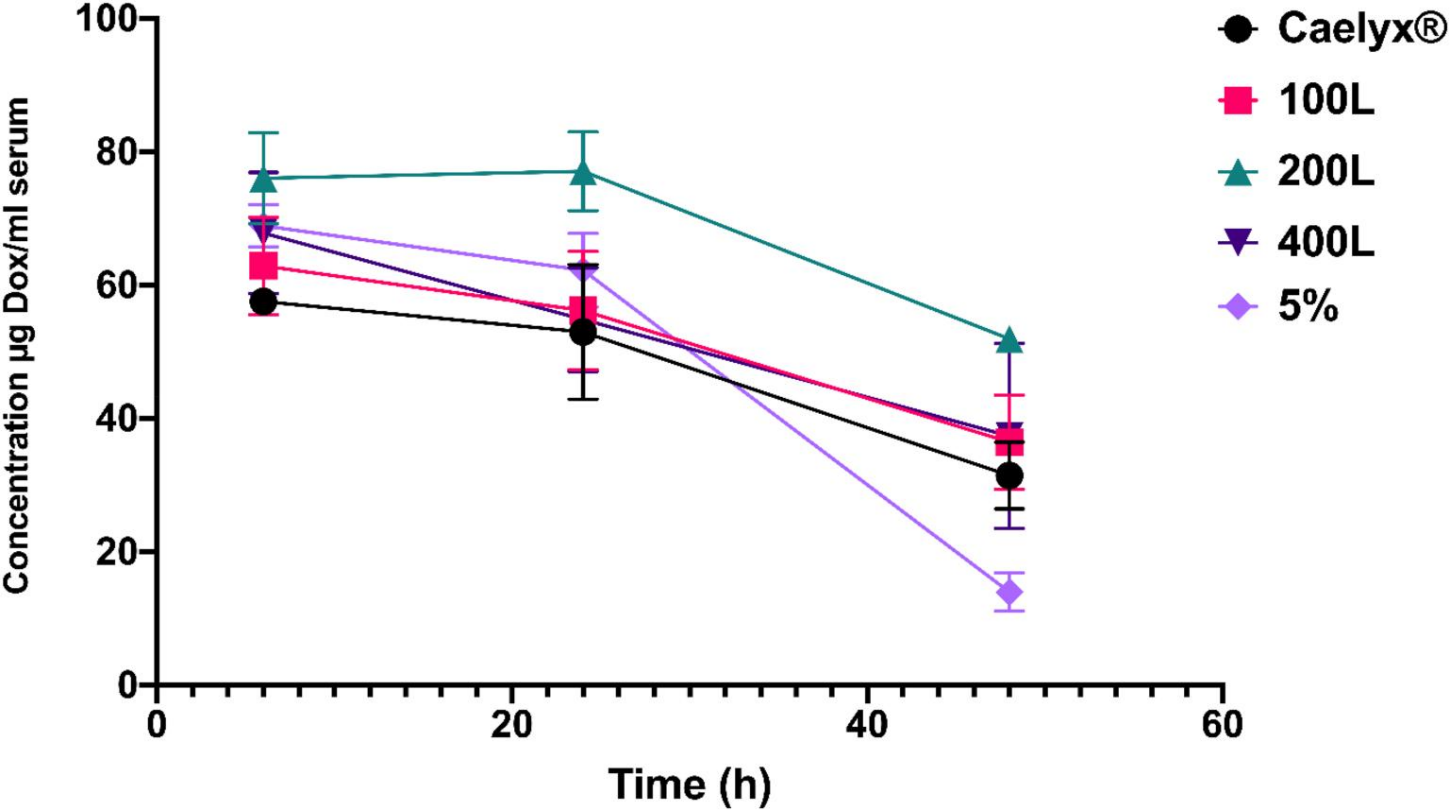
The biodistribution of the pre‐and post‐inserted formulations and Caelyx® at different time points in the blood, in healthy mice after a single dose of 10 mg/kg administered iv. Data expressed as mean ± SEM.

In order to reach a comprehensive assessment of the pre‐ and post‐inserted liposomal formulations' biodistribution, the analyses were performed in the main organs, including blood serum, brain, liver, spleen, kidney, lung and heart. The analyses were done in the main organs, including blood serum, brain, liver, spleen, kidney, lung, and heart, in order to get a comprehensive assessment of the pre‐ and post‐inserted liposomal formulations' biodistribution. As shown in Figure 3, the DOX concentration in blood remained relatively stable in the first 24 h and then dropped dramatically in the following hours, with the 5% pre‐inserted formulation showing the most dramatic drop. In general, however, there was no substantial difference in blood concentrations between Caelyx^®^ and the targeted formulations. The gradual decay in blood concentration of DOX over time enhances the trafficking of DOX through BBB to tumour site.

**FIGURE 4:**
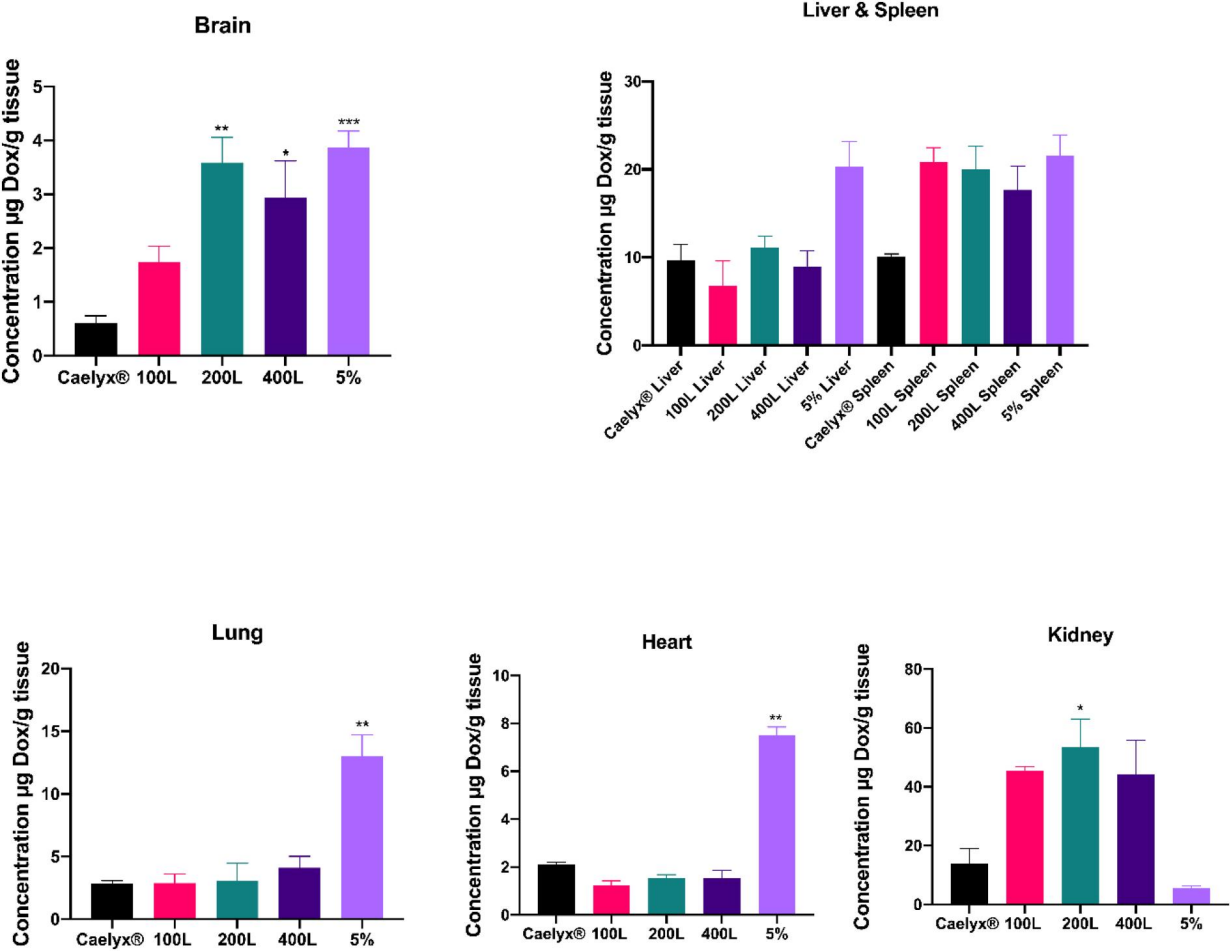
The biodistribution of the pre‐ and post‐inserted formulations and Caelyx® in brain, liver and spleen, lung, heart, and kidney, in healthy mice after a single dose of 10 mg/kg administered iv. Data expressed as mean ± SEM.

Moreover, in comparison to the pre‐ and post‐inserted formulations, Caelyx^®^ had the lowest amount in the brain (under 1 μg/ml), according to the brain data analysis (Figure 4). In comparison to Caelyx^®^, the 200L, 400L, and 5% formulations showed the greatest concentrations in the brain, with substantially different levels. The 5% level was slightly higher than that of the 200L and 400L formulations (Figure 4).

In the liver (Figure 4), none of the post‐inserted formulations made a significant difference, while the 5% was twice more than other formulations in this organ. Furthermore, after 48 h, the concentration of the pre‐ and post‐inserted formulations in the spleen was higher than Caelyx^®^. At this time, the increasing levels were statistically significant for the 100L, 200L, and 5% pre‐inserted formula (*p* < 0.05).

Figure 4 shows that the 5% pre‐inserted formulation accumulated in the lungs' capillaries in large amounts after injection (*p* < 0.01), while the amount of all post‐inserted formulations in this organ (approximately 3–4 g/ml) did not differ significantly.

Furthermore, the amount of Caelyx^®^ in the heart after 48 h was barely higher than the post‐inserted formulations but much lower than the 5% pre‐inserted formulation (*p* < 0.01). In addition, no statistically significant difference in heart concentrations was found between Caelyx^®^ and the post‐insertion formulations.

In contrast, DOX accumulation in the kidney of mice given Caelyx^®^ and 5% pre‐inserted formulations (less than 20 g/ml) was found to be less than the post‐inserted formula.

**FIGURE 5:**
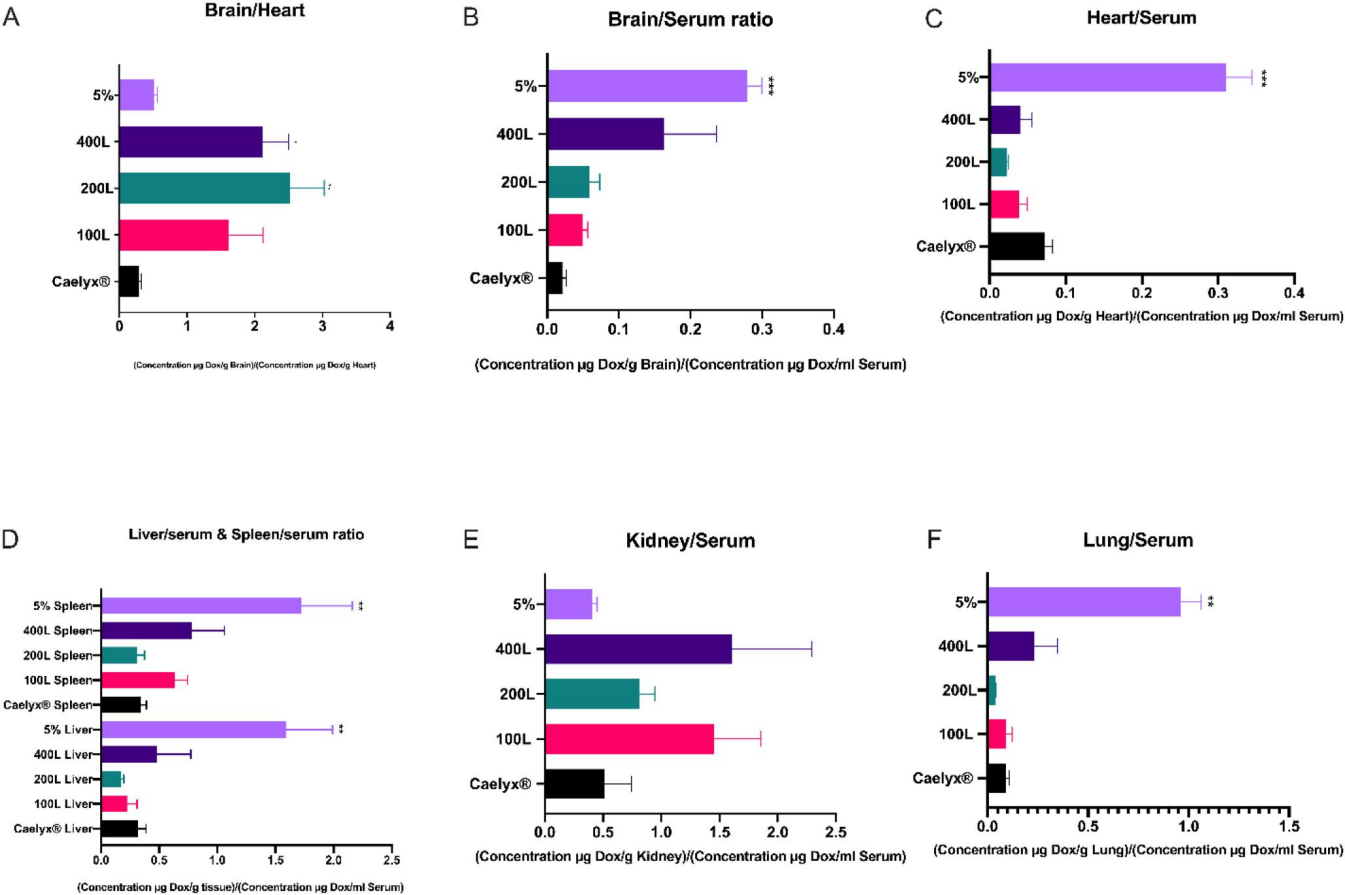
DOX concentration ratio in tissues to the concentration of DOX in serum (b, c, d, e, f). The comparison between DOX concentration in the brain and heart (a) presents the efficacy of the formulations compared to their potential cardiotoxicity. Results are expressed as Mean ± SEM.

The analysed data of the brain/heart ratio that shows efficacy versus potential cardiotoxicity (Figure 5a), established that the post‐inserted formulations accumulated significantly more in the brain than in the heart, possibly resulting in cardiotoxicity. The brain/heart ratios of the 200L and 400L formulations were 2.52 and 2.11, respectively, significantly higher than Caelyx^®^ and 5%.

Furthermore, the 400L formulation had the highest brain/serum ratio among the post‐inserted formulations, followed by 200L, 100L, and Caelyx^®^. Nonetheless, they did not differ significantly from one another. In comparison to the post‐inserted formulations and Caelyx^®^, the 5% pre‐inserted formulation had the highest brain/serum ratio (*p* < 0.001). Furthermore, because the formulations' brain/serum ratio was greater than 0.04 (0.05, 0.06, 0.16, and 0.3 for 100L, 200L, 400L, and 5%, respectively), the pre‐ and post‐inserted formulations can be deemed ‘brain‐penetrant’ [42, 43].

Caelyx^®^ had a heart/serum ratio of 0.07, which was 2.3, 3.5, and 1.75 times higher than the 100L, 200L, and 400L post‐inserted formulations, respectively, according to Figure 5c. Furthermore, the 5% pre‐inserted formulation had a substantially higher heart/serum ratio (0.3) than the other post‐inserted formulations and Caelyx^®^ (*p* < 0.001).

Figure 5d shows that the 5% pre‐inserted formulation had the highest liver/serum and spleen/serum ratios, with a significant difference over Caelyx^®^ for both ratios (*p* < 0.05). It was followed by the 400L formulation, which measured 0.5 in the liver and 0.8 in the spleen, although there was no noticeable difference between the post‐inserted and Caelyx^®^ formulations. Furthermore, the kidney/serum ratio showed the highest 400L accumulation and the lowest 5% pre‐inserted formulation accumulation in this organ. Finally, the lung/serum ratio showed the largest accumulation of the 5% pre‐inserted with a significant difference (*p* < 0.01) over Caelyx^®^, and less accumulation of post‐inserted treatment arms in the lungs.

It is noteworthy that the DOX concertation in different organs of the mice received post‐inserted formulations was as follows: kidney > spleen > liver > lung > brain > heart, which was not significantly different in the three post‐inserted formulations. A remarkable finding in the biodistribution analysis was the lowest DOX concentration in the hearts of mice treated with post‐inserted formulations compared to the mice treated with pre‐inserted formulation and Caelyx^®^. In contrast, the lowest concentration of DOX was in the brains of mice received Caelyx^®^ and pre‐inserted formulation.

### 3.6 Histological study

**FIGURE 6:**
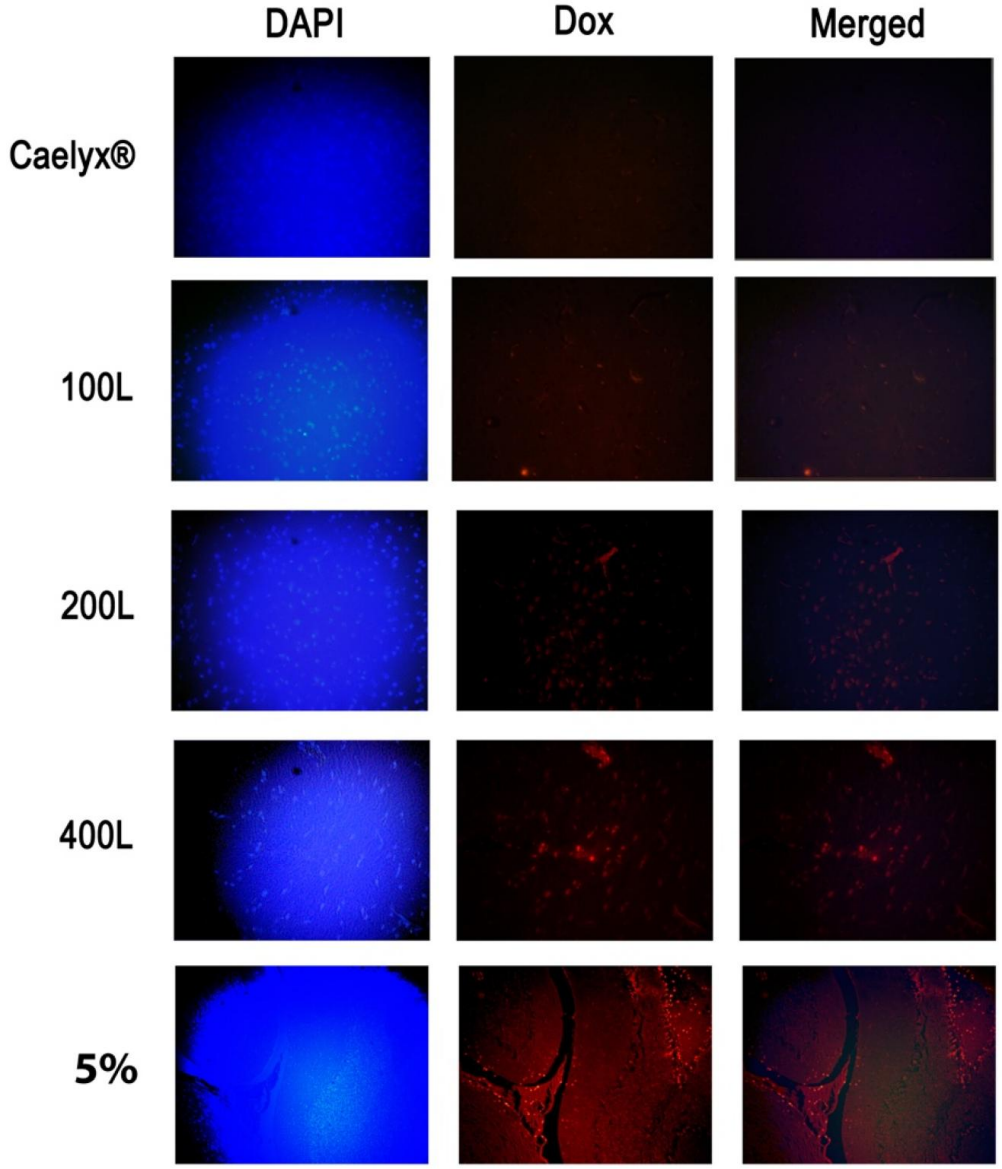
DOX penetration into brain tissue as seen using fluorescence microscopy. DAPI staining was used to stain the sections. Sections are examined at a magnification of ×200.

Using fluorescence microscopy, Figure 6 indicates the degree of DOX penetration into brain tissue.

The 5% pre‐inserted formulation was shown to accumulate the most within the brain tissue, followed by the 400L, 200L, and 100L formulations. Caelyx^®^, on the other hand, showed no evidence of considerable brain entry.

## 4 DISCUSSION

Several researches have looked into the incorporation of targeted ligands on liposomes using a post‐insertion technique [44, 45, 46, 47]. The post‐insertion method is one of the fastest, most efficient, and cost‐effective strategies for designing innovative targeted drug delivery systems, according to previous works [48]. In this study, we used the post‐insertion approach to add the 100, 200, and 400 GSH targeting ligands to the liposomes' surface, resulting in three distinct glutathione decorated PEGylated liposomal DOX.

Furthermore, the 5% pre‐inserted GSH PEGylated liposomal DOX was made by incorporating DSPE‐PEG2000‐Maleimide‐GSH into the Caelyx^®^ by the pre‐insertion process, according to 2B3‐101, which was designed as a brain targeted DOX product.

This research looked into three key objectives, which are outlined below. To begin, using post‐insertion as a more convenient, repeatable, and quick method of incorporating targeted ligands into nanoliposomes. Second, we attempted to take advantage of the liposomal surface's minimum amount of targeting ligands, as opposed to the 2B3‐101 (or 5% in this study), which has over 40 times more ligands on its surface. Using the minimal density of targeting ligands on its surface, the post‐inserted formula was able to achieve the same efficacy with reduced cardiotoxicity, which is more desirable and practical. By including a three‐amino‐acid spacer as well as PEG3400, the third goal was to improve post‐inserted delivery systems while increasing GSH exposure to BBB receptors. As a result, the PEG3400‐GSGGCE formulations were created, and their efficacy and capacity to cross through the BBB were compared to 5% pre‐inserted formulations and Caelyx^®^.

The first experiment involved conjugating GSH and PEG2000 before the pre‐insertion, which was validated by TLC and HPLC to ensure that the connection was made precisely. In the second experiment, before the post‐insertion, GSGGCE and DSPE‐PEG3400 were conjugated, and HPLC was utilised to validate this attachment. Furthermore, the size of the formulations was measured before and after insertion to determine that they were the right size for an intravenous drug delivery system reaching the brain's target region. The formulations were larger than Caelyx^®^, which could indicate that the targeted ligand was incorporated onto the liposome surface. Due to increased GSH‐PEG loadings, the 5% had the most nanometres in size and diameter. Because the GSGGCE targeting ligands were coupled to the PEG3400, the particle size of the post‐inserted GSGGCE‐PEG3400 was slightly larger than Caelyx^®^. The particle homogeneity was validated by the PDI of less than 0.2 [49]. Due to the negative charge of the DSPE‐PEG and GSH, the pre‐ and post‐inserted formulations were more negative than Caelyx^®^ [50]. The post‐inserted formulations' encapsulation efficacy was greater than 95%, revealing that the post‐insertion procedure had no major impact on encapsulation [51].

According to Figure 2, the post‐inserted formulations containing PEG3400 had a faster release percentage than the 5% pre‐inserted formulations and Caelyx^®^ containing PEG2000. The instability of the post‐inserted formulations was probably caused by the physicochemical and surfactant properties of PEG3400 [52, 53]. Due to the presence of concentrated targeting ligands on the liposome's surface, the release of the 5% pre‐inserted formulation was greater than that of Caelyx^®^. Consequently, in simulated acidic tumour microenvironments, the targeted formulations are not considered stable. For all pre‐ and post‐inserted formulations, the release profile rose in the first 10 h and remained consistent over the next several hours. Over a 24‐h period, the Caelyx^®^ released less than 2% of its DOX, whereas the post‐inserted nanostructures released the most DOX. This discovery is consistent with the findings of the study, which show that the lipid composition of the formulations has an impact on drug release during blood circulation as well as the stability of the liposomal bilayer [54].

The formulations were evaluated in vivo in healthy NMRI mice for biodistribution to check if the designated targeted liposomes with GSGGCE, could cross the BBB. In previous studies, tumoral mice were used while brain tumours produce inflammation and alter BBB permeability, allowing the enhanced permeability and retention effect to increase medicine intratumoral concentrations. In healthy non‐tumoral mice, the most probable mechanism to pass through the BBB is receptor‐mediated transcytosis [55, 56].

The distribution of the medications in the blood showed that their concentration decreased after 2 days, possibly due to their accumulation in other tissues, particularly the liver and spleen. This result is consistent with prior research, which found that drug distribution into the blood was dramatically reduced after 2‐ or 3‐days post‐injection [24, 28, 57]. The higher distribution of pre‐ and post‐inserted formulations in the liver and spleen seemed to be due to the presence of the reticuloendothelial system (RES), which was able to rapidly take up the formulations and resulted entrapment in the mentioned organs. Elevated diameters and negative charge of targeted formulations cause further RES involvement [58, 59].

All of the targeted formulations had higher amounts in the brain than the Caelyx^®^, as shown in Figure 4, demonstrating that GSH is one of the most successful targeting ligands to pass through the BBB [60].

Similarly, the brain/serum ratio revealed that, as compared to serum concentration, the brain concentration of the post‐inserted PEG3400‐GSGGCE liposomal formulations grew gradually as the number of GSH ligands increased, demonstrating the proper effect of GSH in brain drug delivery.

As a result, the post‐inserted formulations can be classified as ‘brain‐penetrant’, with a brain/serum ratio of greater than 0.04. Additionally, histological images demonstrated that the 5% pre‐inserted formulations had acceptable brain penetration, followed by 400L, 200L, and 100 L, as previously described (Figure 6). The importance of ligand numbers should be considered. The z‐average results revealed that as the number of ligands rose, the size of the post‐inserted formulations grew slightly, resulting in higher RES uptake and faster serum clearance. The increased distribution of post‐inserted formulations to the liver, and spleen, however, did not affect drug delivery to the brain. As a result, these formulations can be considered potentially beneficial medications in the treatment of brain tumours.

The accumulations of the pre‐ and post‐inserted formulations were higher than Caelyx^®^ within the lungs' capillaries due to the presence of GSH receptors in the lungs [61, 62, 63]. Caelyx^®^ heart distribution was nearly identical to the post‐inserted formulations, as shown in Figure 4. The interactions between the liposomes and cardiac GSH receptors have increased since a larger concentration of GSH was used in generating the 5% pre‐inserted formulations. As a result, the 5% heart distribution was significantly higher than that of the post‐inserted and Caelyx^®^ (*p* < 0.01). In comparison to 5% and the Caelyx^®^, the brain/heart ratio demonstrated that the post‐inserted formulations overcome the risk of probable cardiotoxicity through advantageous penetration into the brain and lowered heart drug accumulation. In other words, when compared to the Caelyx^®^, the DOX content in the post‐inserted formulations was significantly lower in the heart and significantly greater in the brain tissues, showing a more targeted function of these targeted formulations. The brain/heart ratio dropped with the 5% pre‐inserted nanoliposomes, despite the presence of significant levels of DOX in the brain tissue. This was due to a similarly higher concentration of DOX in the heart. According to the aforementioned explanations, the 5% formula may not be as effective as the post‐inserted 400L with identical brain concentrations and a reduced heart distribution. The 5% pre‐inserted formulation showed the highest brain/serum and heart/serum ratios when compared to the other formulations. As a result, while this formulation had a normal concentration in the brain, its large accumulation in the heart may have induced cardiotoxicity.

The higher concentration of post‐inserted formulations in the kidney may be due to the transfer of DOX released from post‐inserted formulations with the greatest release profile from blood to the kidney, which is part of the excretory system, after 24 h post‐injection.

Although previous studies have shown that conventional GSH ligand incorporation improves DOX brain biodistribution, this study demonstrated that using the post‐insertion technique can be more efficient, easy, and cost‐effective. The findings of our animal investigation are consistent with the findings of other studies, which found that the modification of liposomal DOX with different targeting ligands improved therapeutic efficacy [64, 65, 66].

This study found that, despite Caelyx^®^ and a 5% pre‐inserted PEG2000‐GSH formulation, using a spacer, PEG3400 in the preparation of PEG3400‐GSGGCE liposomal formulations could effectively increase the ability of these drugs to cross the BBB, as demonstrated in biodistribution and histological studies, and thus reduced adverse effects such as cardiotoxicity. To maximise the potential of GSH‐targeted liposomal DOX, a number of areas merit further exploration, including optimisation of the number of ligands, evaluation of different spacers and assessment of efficiency in tumour inoculated models.

## 5 CONCLUSION

In this study, the distribution of the PEG3400‐GSGGCE liposomal formulations into the brain using the post‐insertion method was evaluated, and compared with that of the 5% pre‐inserted PEG2000‐GSH formulation. It was shown that the post‐insertion approach was more economic, efficient, and followed an easier method of preparation compared to the pre‐insertion. These novel formulations could interestingly increase the brain concentration of accumulated DOX. According to the evaluated brain/heart biodistribution ratio, the PEG3400‐GSGGCE liposomal formulations' concentration was lower in the heart and higher in brain tissues, compared to the 5% pre‐inserted PEG2000‐GSH formulation and Caelyx^®^, and there was not any considerable difference between the post‐inserted formulations' distribution in different organs. The results of this study can be compared to the PEG2000‐GSH formulation prepared according to 2B3‐101, and our previous study in which we used PEG2000 for the post‐inserted GSH‐Caelyx® formulations, in terms of efficacy and biodistribution.

## AUTHOR CONTRIBUTIONS

**Amin Mehrabian**: Methodology; Writing – review & editing. **Saba Dadpour**: Methodology; Writing – original draft; Writing – review & editing. **Mohammad Mashreghi**: Formal analysis. **Javad Zarqi**: Formal analysis; Methodology. **Anis Askarizadeh**: Methodology. **Ali badiee**: Writing – review & editing. **Leila Arabi**: Writing – review & editing. **Seyedeh Alia Moosavian**: Writing – review & editing; Supervision; Validation. **Mahmoud Reza Jaafari**: Supervision; Validation; Project administration.

## CONFLICT OF INTEREST

The authors declare that they have no known competing financial interests or personal relationships that could have appeared to influence the work reported in this paper.

## PERMISSION TO REPRODUCE MATERIALS FROM OTHER SOURCES

None.

## Supporting information

Supporting Information S1

## Data Availability

The data that support the findings of this study are available on request from the corresponding authors.
