# Supplementary material for: The comparison of biodistribution of glutathione PEGylated nanoliposomal doxorubicin formulations prepared by pre‐insertion and post‐insertion methods for brain delivery in normal mice
Source: IET Nanobiotechnol. 2023 Jan 3;17(2):112–24. doi: 10.1049/nbt2.12111 (PMC10116028; doi:10.1049/nbt2.12111)
Supplement: Supplementary file 1 — Supporting Information S1 [file NBT2-17-112-s001.docx]

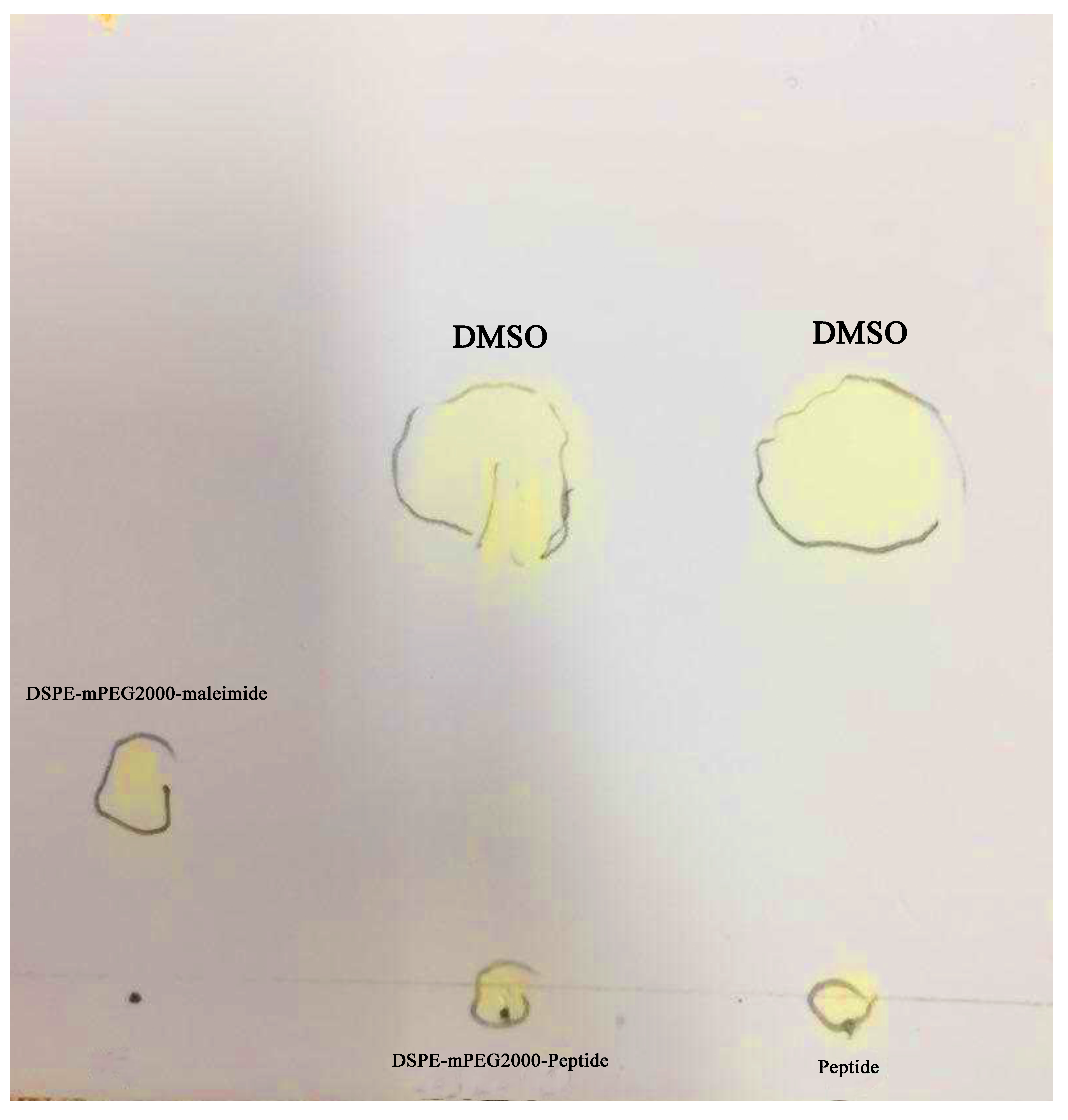


**Figure S1.** TLC chromatography. The PEG, and P+P are abbreviations for free PEG_2000_-MAL., PEG2000-GSH complex, and free GSH peptide, respectively. The chromatograph shows no similar spots in the (P+P) spot area that demonstrates the end of the conjugation reaction.


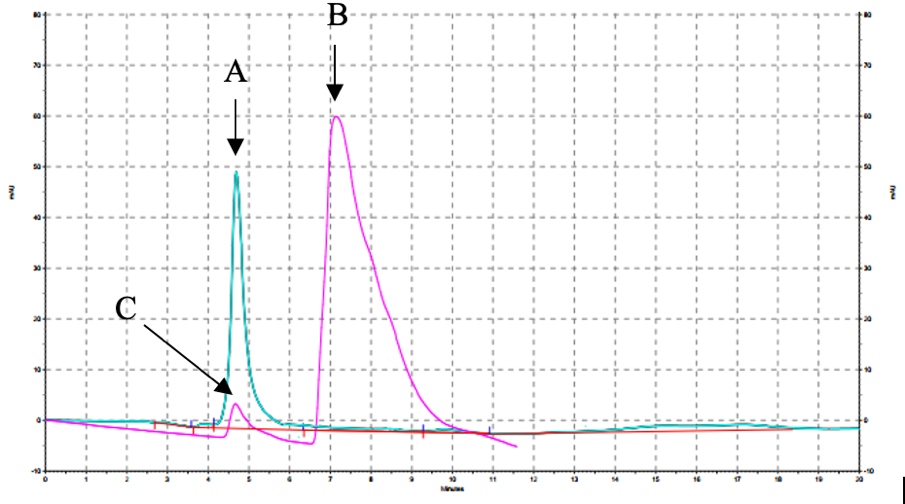


**Figure S2.** HPLC chromatography as the complex linking assay. (A) is the result of the first injection which represents the free peptide that was eluted with a retention time of ∼4.7 min. (B) represents the PEG2000-GSH filtrate and (C) represents the free peptide at the exact retention time as the free GSH which was injected to verify and identify the complex from the free peptide.


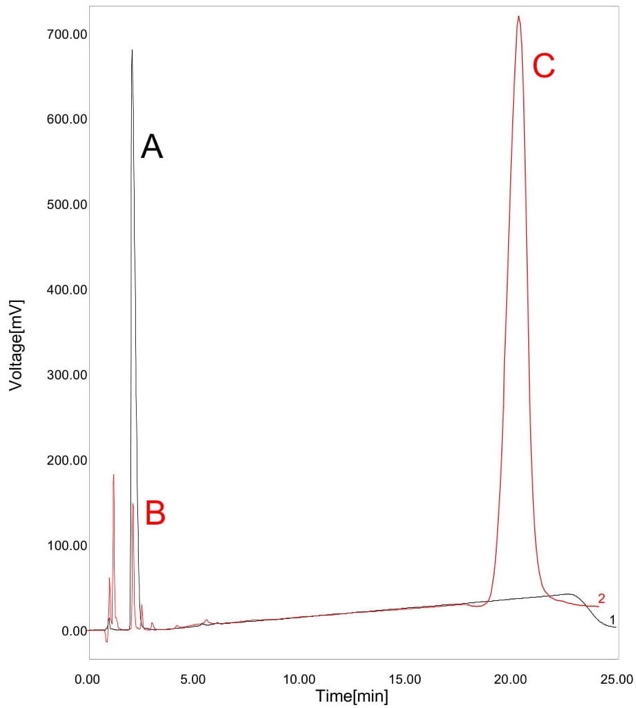


**Figure S3.** The analytical HPLC chromatogram. (A) represents the first injection of free peptide (GSH); (B) shows the remained and unreacted peptide after the reaction; (C) indicates the final product (PEG3400-GSGGCE complex).
